# Recognition to repair: Managing reservoir complications in inflatable penile prosthesis surgery

**DOI:** 10.1002/bco2.70246

**Published:** 2026-07-07

**Authors:** Sarah Michael, Peter Grice, Adam Oumeziane, Michael George, David Riding, Deena Harji, Theodora Stasinou, Ian Pearce, Vaibhav Modgil

**Affiliations:** ^1^ Manchester Andrology Research Collaborative Manchester Royal Infirmary Manchester UK; ^2^ Faculty of Biology, Medicine, and Health University of Manchester Manchester UK; ^3^ Manchester University NHS Foundation Trust Manchester UK; ^4^ Manchester Vascular Centre Manchester Royal Infirmary Manchester UK; ^5^ Department of Colorectal Surgery Manchester University NHS Foundation Trust Manchester UK

**Keywords:** penile implant, penile implant intraoperative complications, penile prosthesis, reservoir injuries, reservoir‐related complications

## Abstract

**Introduction:**

Reservoir placement during three‐piece inflatable penile prosthesis (IPP) implantation is the step most commonly associated with serious intraoperative complications. The reservoir is traditionally positioned within the space of Retzius, where it lies in close proximity to the bladder, bowel and major pelvic vessels. Although injuries are uncommon, damage to these structures can result in significant morbidity, particularly in patients with prior pelvic surgery, radiotherapy or distorted pelvic anatomy. As prosthetic surgery expands to more complex patient populations, surgeons must be prepared to recognise and manage these complications when they occur.

**Results:**

This review focuses on the intraoperative injuries associated with reservoir insertion during IPP surgery and provides a practical framework for their recognition and management. Bladder, bowel and vascular injuries are discussed with emphasis on intraoperative warning signs, methods of confirmation and immediate management strategies. Key principles of repair, haemorrhage control and involvement of specialist surgical teams are outlined. Guidance is also provided on intraoperative decision‐making, including when reservoir relocation, staged implantation or procedure abandonment may be appropriate to minimise the risk of prosthesis infection and other complications. Alternative reservoir placement techniques are briefly discussed in the context of risk reduction, particularly for patients with hostile pelvic anatomy. However, the primary aim of this review is to provide pragmatic guidance for surgeons confronted with intraoperative complications during reservoir insertion.

**Conclusions:**

Early recognition, prompt management and a structured approach to intraoperative decision‐making are essential to minimise morbidity and preserve the favourable functional outcomes associated with IPP surgery.

## 1 INTRODUCTION

Erectile dysfunction (ED) is a common condition defined as the persistent difficulty in achieving or sustaining an erection that is adequate for sexual activity.^1^ Its prevalence increases with age and is strongly associated with cardiovascular disease, diabetes and psychological comorbidities. Population‐based studies estimate that 20%–25% of men in their 60s experience moderate to severe ED, rising to nearly 80% in those over 80.^2^ With an aging and multimorbid population, the demand for durable and definitive treatment options is expected to rise.^3^

Although first and second line treatment options such as pharmacological therapies, vacuum devices and intracavernosal injections have been shown to be efficacious, many men will fail to achieve reliable long‐term success with this.^4^ For these patients, penile prosthesis implantation remains the most definitive treatment, with reported satisfaction rates exceeding 80%–90%.^5^ Among available devices, the three‐piece inflatable penile prosthesis (IPP) is regarded as the gold standard.^6^ Since Dr Scott's first modern design in the 1970s,^7^ refinements in the device have ensured high satisfaction rates,^5^ as well as positive functional outcomes and long‐term efficacy.^8^ The modern three‐piece IPP is made up of three key components: a scrotal pump, a fluid reservoir and two inflatable cylinders.^9^

Despite these advantages, IPP implantation is not without risk. Reservoir placement represents the most technically hazardous step of the procedure. The reservoir is traditionally placed within the space of Retzius (SOR), an anatomically constrained region in close proximity to the bladder, bowel and major pelvic vessels. Although major injuries are uncommon, when they occur, they may be severe and associated with significant morbidity.^10^, ^11^ Increasing numbers of patients with prior pelvic surgery or radiotherapy present with distorted anatomy, raising the risk of iatrogenic complications.^12^, ^13^ In response to these risks, alternative (ectopic) reservoir placement techniques have been increasingly adopted.

The aim of this review is to summarise the literature around reservoir placement and iatrogenic injuries and provide a practical framework for recognition and management of bladder, bowel and vascular injuries with the goal of providing pragmatic guidance for andrologists undertaking this procedure. Where high‐quality comparative data are lacking, recommendations reflect the expert practice of high‐volume implanters and are intended to guide intraoperative decision‐making rather than serve as prescriptive algorithms.

## 2 CONVENTIONAL PLACEMENT AND RISKS OF RESERVOIR INSERTION

The three‐piece IPP functions by transferring saline from the reservoir to corporal cylinders via a scrotal pump, producing rigidity sufficient for sexual activity including penetrative intercourse.^7^ The reservoir, typically containing 65–125 mL of saline, is conventionally placed within the SOR. The SOR is a potential retropubic space bordered anteriorly by the pubic symphysis, posteromedially by the bladder and laterally by the external iliac vessels. This location provides a low‐pressure environment that minimises the risk of auto‐inflation while offering excellent concealment.^14^

Penile prosthesis insertion may be performed via infrapubic, penoscrotal or less commonly subcoronal approaches. The infrapubic approach allows more controlled access to the retropubic space, whereas the penoscrotal approach typically relies on blind passage.^15^ A less frequently used subcoronal approach requires an additional incision for reservoir placement.^16^ Ultimately, approach selection depends on surgeon experience, training and patient‐specific factors.

Despite these functional advantages, the SOR is an anatomically hazardous zone. Radiographic and cadaveric studies highlight the close proximity of critical structures: the bladder lies posterior to the dissection plane around 5–8 cm away, the external iliac vein averages only 2.6–3.1 cm lateral to the reservoir pocket and tributaries such as the inferior epigastric and external pudendal veins traverse the operative field.^17^ In patients with prior pelvic surgery or adhesions, loops of small bowel or sigmoid colon may descend into the pelvis, further narrowing the safe operating field.^18^

Although the reported incidence of visceral or vascular injury during conventional placement is low,^19^ complications can be severe with significant morbidity. This risk is greatest in men with a history of radical prostatectomy, cystectomy, pelvic radiotherapy or mesh hernia repairs, where scarring obliterates natural planes.^12^, ^13^ The surgical approach also matters. Blind reservoir passage through the penoscrotal route carries a higher likelihood of bladder injury than infrapubic placement, which permits more direct and controlled entry into the retropubic space.^15^, ^20^

Precautionary strategies include ensuring bladder decompression either through catheterisation or, less reliably, having the patient void preoperatively, adopting the Trendelenburg position to displace bowel, and meticulous blunt finger dissection rather than forceful instrument passage.^17^ Nevertheless, even with optimal technique, distorted anatomy such as those described above can render SOR placement hazardous and this has fuelled increased use of alternative, ectopic reservoir sites.

## 3 ALTERNATIVE (ECTOPIC) RESERVOIR PLACEMENT

In response to these risks, ectopic reservoir placement techniques have been developed to deliberately avoid the retropubic space. These approaches aim to reduce the risk of bladder, bowel and vascular injury while maintaining adequate concealment and patient satisfaction.^19^, ^21^, ^22^ For new implanters, ectopic placement should be viewed as a primary risk‐reduction strategy in patients with hostile pelvic anatomy rather than a salvage technique. A comparative overview of reservoir sites is summarised in Table 1.

**TABLE 1:** An overview of reservoir sites and associated features.

Reservoir position	Adjacent structures	Advantages	Disadvantages	Indications
Conventional (retropubic)	Posterior to transversalis fascia, inferior to peritoneal reflection, lateral to bladder External iliac vessels are found lateral	Most commonly performed Reliable cosmetic and practical outcomes Low‐pressure space with low risk of auto‐inflation	Risk of visceral injury Hernia risk	Primary implant in patients without prior pelvic surgery or irradiation
High extraperitoneal (ectopic)	Posterior to transversalis fascia, anterior to peritoneum	Low‐pressure space with low risk of auto‐inflation Accessible via single infrapubic incision Excellent concealment	Potential reservoir palpability Reservoir herniation	Complex pelvic anatomy Revision implants with obliterated standard spaces Primary implantation via infrapubic approach
High submuscular	Between transversalis fascia and posterior rectus sheath, deep to rectus muscle	Extraperitoneal Not located next to critical pelvic structures	Potential reservoir palpability Less familiar and more technically challenging	Complex pelvic anatomy or salvage/reimplantation cases
Subexternal oblique	Deep to external oblique muscle Superficial to internal oblique muscle	Extraperitoneal Not located next to critical pelvic structures	Novel technique with limited outcome data May be palpable	Complex pelvic anatomy Revision implants with obliterated standard spaces
Lateral retroperitoneal	Anterior to psoas muscle Posterior to transversalis fascia lateral to rectus sheath and external iliac vessels	Low‐pressure space with low risk of auto‐inflation Not located next to critical pelvic structures	Less familiar and more technically demanding Potential for suboptimal position	Complex pelvic anatomy, and limited other options
Peritoneal	Within the peritoneal cavity, adjacent to bowel or bladder	Last resort in obliterated planes	High risk of bowel obstruction, adhesions or visceral injury Technically demanding and invasive High risk of auto‐inflation	Failed prior placement
Subcutaneous	Deep to the subcutaneous fat and skin	Minimally invasive	Highly visible Palpable and mobile reservoir Superficial placement may compromise function	Failed prior placement Restricted to obese/highest risk patients

The high submuscular (HSM) technique is the most widely adopted ectopic placement. The reservoir is positioned beneath rectus abdominis but superficial to the transversalis fascia, creating an extraperitoneal pocket that avoids the bladder and iliac vessels.^17^, ^23^ Single and multicentre experiences demonstrate excellent safety, including in men with prior pelvic surgery, with very low rates of visceral or vascular injury.^24^, ^25^ Reported patient satisfaction and reservoir‐related event rates are favourable, with palpability, herniation or auto‐inflation uncommon when pockets are properly developed.^24^, ^26^

Placing the reservoir one layer deeper between the fascia transversalis and the anterior aspect of the peritoneum, (high extraperitoneal) offers a low‐pressure highly concealable option favoured during infrapubic prosthesis placement.^27^ This theoretical space is developed through placement via the external inguinal ring with excellent patient satisfaction and low rates of complications, for example, reservoir herniation, auto‐inflation and visceral damage. Positioning the reservoir away from the bladder also negates the need for peri‐operative catheterisation.

The subexternal oblique (SEO) approach represents a further refinement designed to avoid pelvic entry altogether. By positioning the reservoir between the external and internal oblique muscles, this technique theoretically eliminates the risk of bladder or iliac vessel injury while maintaining concealment.^19^, ^28^ Although outcome data are limited to small series, early reports suggest that the approach is feasible and safe, particularly in revision cases where deeper extraperitoneal planes are obliterated.^21^, ^28^

Lateral retroperitoneal placement has also been described as a means of bypassing scarred or hostile midline tissues. Positioning the reservoir anterior to the psoas muscle and posterior to the transversalis fascia maintains a low‐pressure environment while avoiding proximity to the bladder and major pelvic vessels. Although technically more demanding and less familiar, published series report low complication rates and satisfactory outcomes in patients with complex pelvic anatomy.^24^, ^29^

Intraperitoneal and subcutaneous reservoir placements are less commonly performed and are generally reserved for salvage situations. Intraperitoneal placement exposes the reservoir to bowel and bladder, with risks of adhesion formation, obstruction and visceral injury.^19^, ^22^ Subcutaneous placement avoids pelvic entry but carries drawbacks including visibility, palpability and migration and is typically limited to very obese patients where concealment is possible.^21^, ^30^ Consequently, both approaches should be considered last‐resort options rather than routine strategies.

**FIGURE 1:**
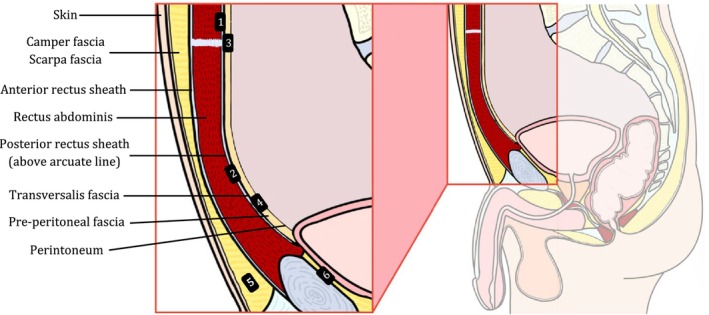
Schematic illustration of locations for implantation of IPP reservoir, midline sagittal view. Original figure with concept inspired and modified from work of Dick et al.^31^ (1) High submuscular and (2) submuscular, located deep to rectus abdominis. (3) High extraperitoneal and (4) extraperitoneal, located deep to the transversalis fascia and superficial to the peritoneum. (5) subcutaneous, located in subcutaneous tissue. (6) Space of Retzius, located in the retropubic (also known as prevesical) space.

**FIGURE 2:**
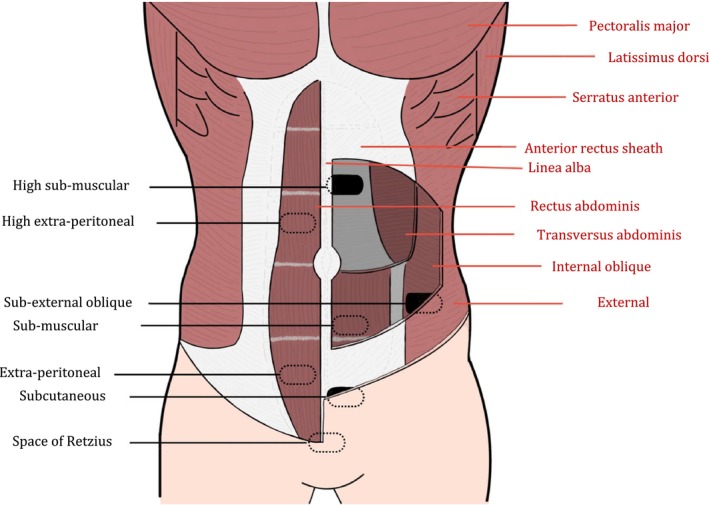
Schematic illustration of locations for implantation of IPP reservoir, anterior view with key layers of abdominal wall labelled.

Taken together, ectopic reservoir placement techniques represent a direct response to the complication profile of conventional retropubic placement. HSM placement has the strongest evidence base and should be considered the first‐line ectopic option, with SEO and lateral retroperitoneal approaches providing valuable alternatives in complex revision cases. Representative schematics are shown in Figures 1 and 2.

## 4 SPECIFIC INJURIES TO KEY STRUCTURES RELATED TO RESERVOIR PLACEMENT

### 4.1 Bladder injury

Bladder perforation is the most frequently reported visceral complication associated with reservoir placement and remains a defining risk of accessing the SOR. Although the reported incidence in contemporary series is low, typically around 1%–2%, the clinical consequences can be significant, often necessitating intraoperative repair, prolonged catheterisation or modification of the implantation strategy.^11^, ^32^, ^33^ The true incidence is likely underestimated, as much of the literature is retrospective and some injuries are recognised only postoperatively following presentations with haematuria, urinary leakage, urinoma or prosthesis infection.^34^, ^35^

Early recognition is critical. Intraoperative warning signs include sudden loss of resistance during pocket development, clear fluid welling into the operative field, visualisation of a catheter balloon or unexpected gross haematuria.^34^, ^36^ When suspicion arises, further dissection should cease immediately. Intraoperative cystoscopy or an on‐table cystogram provides a rapid and reliable method to confirm the diagnosis and distinguish between intraperitoneal and extraperitoneal injury.^36^

Confirmed extraperitoneal bladder injuries can usually be managed conservatively with urethral catheter drainage, broad‐spectrum antibiotics and relocation of the reservoir to the contralateral side or an ectopic position if appropriate. Catheter drainage is typically maintained for 10–14 days, with cystographic confirmation of healing prior to removal. In contrast, intraperitoneal bladder injuries require formal surgical repair. As standard penile prosthesis incisions provide inadequate exposure, additional access, most commonly via a Pfannenstiel incision or midline laparotomy, is required. The bladder defect should be closed in two watertight layers using absorbable suture, for example, 3–0 vicryl, followed by urethral catheter drainage for approximately 14 days. A suprapubic catheter may be considered in cases requiring extensive repair.^37^ If a laparotomy is performed, the bowel should be assessed to exclude concomitant enteric injury, as adhesions can draw loops of bowel into the retropubic field.^38^ Broad‐spectrum antibiotics should be administered, and a large pelvic drain should be positioned adjacent to the repair to allow for adequate drainage. Laparoscopic and robotic approaches to intraperitoneal bladder repair have been described in case series for urological and general surgical trauma but their use would depend on the availability and expertise of suitably trained urologists.^39^, ^40^

The question then becomes whether to proceed with implantation. When injury is recognised promptly, repair is secure, contamination is minimal and there is no bowel involvement, some series report safe continuation of implantation with relocation of the reservoir to an ectopic site such as the HSM or lateral retroperitoneal space.^34^, ^36^ Where there is gross urine spillage, tenuous repair, irradiated or fibrotic tissue or concern for bowel involvement, the risk of prosthesis infection is substantial. In such circumstances, implantation should be deferred. At this stage of the operation, the corporal dilation, cylinder and pump placement are already complete. To avoid loss of penile length, one option is to keep the cylinders partially inflated and pump in situ with the tubing capped and defer reservoir implantation until bladder healing is confirmed or a malleable implant may be inserted. If there are even greater concerns for infection, the procedure may be abandoned; however, leaving the patient without an implant may result in significant loss of penile length and girth. A malleable device or inflated cylinders would preserve corporal space without requiring a reservoir and can later be exchanged if required once healing is complete.^35^, ^36^ For either of these options, a prolonged course of antibiotics, guided by urine culture results and microbiology input, is recommended.

**FIGURE 3:**
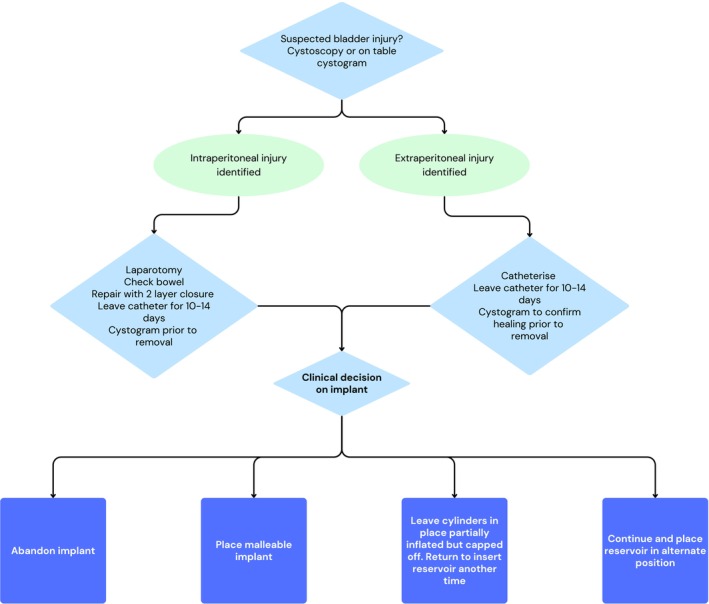
Flow chart detailing steps to take in event of a suspected bladder injury.

Outcomes depend heavily on the timing of recognition. When identified intraoperatively and managed appropriately, bladder injuries generally heal without sequelae, and patients go on to achieve good long‐term prosthesis function. In contrast, delayed or missed perforations can result in urinoma, pelvic abscess, cysto‐cutaneous fistulae, prosthesis infection or explantation. This reality underscores the importance of vigilance, intraoperative MDT decision‐making if possible, preparedness for major repair and a low threshold for confirmation when concerns arise. A simple flow chart to follow in these cases is presented in Figure 3.

### 4.2 Bowel injuries

Visceral gastrointestinal complications associated with penile prosthesis implantation are rare but clinically significant and are most commonly related to reservoir insertion, positioning or long‐term interaction with intra‐abdominal structures.^14^ Acute complications typically arise at the time of reservoir placement due to direct perforation, while delayed complications are more often related to reservoir or tubing migration, erosion, fistulation, obstruction, herniation, infection or adhesion formation.^11^

In the virgin pelvis, the peritoneum holds the small bowel and sigmoid colon superiorly, keeping them away from the retropubic space. However, prior pelvic surgery, radiation therapy or peritoneal violation can distort this arrangement, allowing bowel loops to descend into the operative field. Adhesions formed after such interventions are the critical risk factor, tethering bowel directly into the path of blind dissection.^11^, ^17^

The consequences of missed or delayed recognition are dire. Patients may present postoperatively with peritonitis, sepsis, or abscess formation, often requiring urgent laparotomy and invariably necessitating reservoir removal. These outcomes underscore why bowel injury occupies a place of particular concern among implanters. It is also the clearest demonstration of the rationale for extrapelvic reservoir techniques. HSM and lateral retroperitoneal placements, by avoiding entry into the pelvis, virtually eliminate the possibility of iatrogenic bowel trauma. Indeed, in large contemporary series employing these techniques, no cases of bowel perforation have been reported.^41^, ^42^, ^43^, ^44^ In all scenarios, reservoir placement should be abandoned, as the contamination of the operative field with enteric flora carries an unacceptably high risk of device infection. Case series report patients needing colostomies following bowel perforation due to the severity of the complication.^45^, ^46^

Early recognition is critical. Intraoperative warning signs include unexpected loss of resistance during reservoir pocket development, entry into the peritoneal cavity, feculent contamination, enteric fluid, gas or bleeding inconsistent with standard retropubic dissection. When bowel injury is suspected, further dissection should cease immediately, and early involvement of a general or colorectal surgeon is essential for definitive repair.

When bowel injury is confirmed or strongly suspected, implantation of the reservoir component is contraindicated, and the procedure should be abandoned. Contamination of the operative field with enteric flora confers an unacceptably high risk of prosthesis infection and subsequent explantation. Immediate priorities include control of contamination, administration of broad‐spectrum intravenous antibiotics in accordance with local policy and preparation for definitive surgical repair. Definitive management typically requires primary repair, resection or diversion depending on the site of injury, degree of contamination and patient physiology and may require laparotomy or laparoscopy based on surgical expertise and exposure requirements.

As discussed previously, corporal dilatation will result in subsequent loss of length and girth if no device is left in situ. Following source control and discussion among the operative team and with the aid of senior surgeons, intracorporeal management options are dictated by the degree of contamination and operative findings. In the setting of gross contamination, complete explantation of all prosthetic material represents the safest strategy. Where contamination is minimal, distant from the corporotomy sites and the operative field is judged to be adequately controlled, removal of the reservoir alone with temporary retention of the cylinders and pump may be considered with the cylinders inflated to approximately 50% of their capacity and capped off. Alternatively, a malleable prosthesis can be used as a temporising measure with delayed conversion to a three‐piece inflatable device once healing is complete. In all cases, further implant surgery should be deferred until full recovery.

Delayed or unrecognised bowel injuries may present postoperatively with ileus, peritonitis, pelvic abscess, fistula formation or sepsis.^47^ In patients with recognised risk factors or unexplained postoperative deterioration, a low threshold for investigation with contrast‐enhanced CT of the abdomen and pelvis is warranted. In confirmed cases of delayed perforation, erosion or fistulation, explantation of any prosthetic material is usually required in conjunction with bowel repair or resection.

**FIGURE 4:**
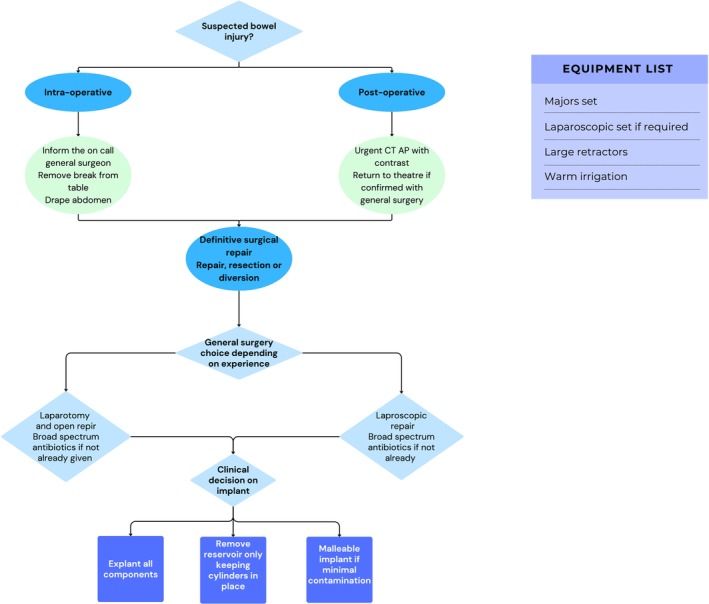
Flow chart detailing steps to take in the event of a bowel injury.

A practical summary of recognition and management is presented in Figure 4.

### 4.3 Vascular injury

Intraoperative vascular injury is a recognised complication of penile implant reservoir insertion. Injuries to proximate vessels should be considered ‘major’ (affecting the iliac vessels) or ‘minor’ (affecting smaller branches such as inferior epigastric, superficial external pudendal or cremasteric vessels), though injury to any vessel may cause significant bleeding.^48^, ^49^ Although most injuries will present themselves intraoperatively, postoperative diagnosis of arterial pseudoaneurysm is possible. An understanding of how to manage vascular injuries is essential for surgeons performing penile implant surgery.

The clinical presentation is typically dramatic, with sudden, brisk bleeding that obscures the operative field and may result in expanding pelvic hematoma or hemodynamic instability. While arterial injury is possible, most reported cases involve venous structures, particularly the external iliac vein or its branches.^33^, ^50^, ^51^, ^52^ Survey data from prosthetic surgeons have identified vascular injury as a major source of intraoperative anxiety and a frequent justification for choosing HSM reservoir placement in men with prior pelvic surgery or mesh herniorrhaphy, where anatomical distortion further narrows the safe operative field.^53^

The first step is to undertake a comprehensive history and examination in the clinic, as this will be useful in establishing whether the patient has had previous arterial or venous surgery. A history of open vascular surgery should be suspected if the patient has either longitudinal or oblique scarring over the femoral triangle. In addition, the femoral vessels are commonly used access sites for endovascular interventions and may induce less obvious (but still significant) perivascular scarring.

If a nonmidline approach is preferred when introducing implant reservoirs, then access via the contralateral side to the previous vascular surgery should be preferred. If the patient has a history of bilateral inguinal vascular surgery, then a midline infrapubic approach may be considered. In the event of major vessel injury, open surgical repair in a scarred field would be more technically demanding and would confer a greater risk of complications (postoperative wound infection, seroma, bleeding and delayed healing for example).

#### 4.3.1 Major vascular injury

If iliac artery or vein injury is suspected during reservoir implantation, then early involvement of vascular surgery teams is essential. It is obvious that vascular trauma may cause bleeding, but iliac arterial injuries are also associated with thromboembolic complications or occlusions. This can cause lower limb ischaemia leading to limb loss or death, mandating expert contribution to the operation at this point.

While waiting for help to arrive, there are several interventions that the operating team can deploy to minimise risk to the patient:

- Manual pressure and/or gauze swab packing around the injured vessel can effectively control bleeding.
- Administration of 1 g of tranexamic acid within 1 h of bleeding onset may significantly reduce the risk of death^54^.
- Activation of the Major Haemorrhage Protocol will ensure the necessary blood products are rapidly available.
- If life‐threatening venous bleeding is present, then the injured vein can be ligated. Ligation of iliac veins is likely to cause lower limb swelling but is a legitimate manoeuvre to save life. ‘Blind’ suturing is not recommended as this can cause a more complex injury to the vessel or can damage adjacent structures.

**FIGURE 5:**
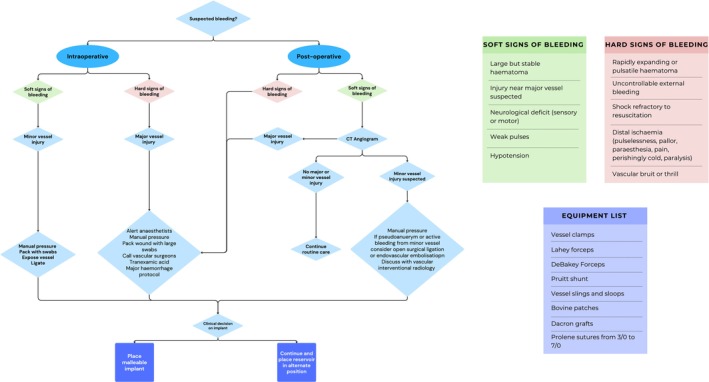
Flow chart detailing steps to take in the event of a vascular injury.

In the event of suspected postoperative bleeding and in addition to the measures above:

- If major vascular injury is suspected postoperatively but the patient is too unstable to undergo safe CT angiography and/or displays hard signs of vascular trauma (see Figure 5), then the patient should be transferred to a hybrid operating theatre if available or a conventional operating theatre if not.
- If major vascular injury is suspected postoperatively but the patient is stable and/or is displaying only soft signs of vascular trauma (see Figure 5), then CT angiography is the imaging modality of choice.

A detailed overview of the vascular repair of iliac injuries is beyond the scope of this paper, but in general terms, the principles of emergency vascular surgery are to resuscitate the patient while gaining proximal and distal vessel control, to repair the injury and to mitigate any thromboembolic or ischaemic complications by restoring limb perfusion. Once bleeding has been controlled and perfusion restored, the patient should continue their resuscitation in a Level 2 or Level 3 bed and can return to theatre for wound inspection and/or repair at a later date, if necessary.

#### 4.3.2 Minor vascular injury

Injury to smaller second‐ or third‐order branches such as the inferior epigastric, superficial external pudendal and cremasteric vessels may cause bleeding, but such injuries do not confer the same risk of thromboembolic or ischaemic complications as major arterial injuries. Accordingly, these vessels can be ligated without consequence, and attempted repair is not indicated. Manual compression and/or gauze swab packing can be deployed to control the bleeding, and blood products may need to be administered as blood loss can still be significant. More extensive surgical exposure of the vessel will aid ligation.

Postoperative pain, swelling, erythema and bleeding through the surgical wound are all recognised signs of inferior epigastric artery pseudoaneurysm. If this is suspected in the postoperative period, then CT angiography is indicated, and consideration should be given to either endovascular embolisation or open surgical ligation.^55^

Once again, a clinical decision is needed on proceeding with implantation. In the event of severe bleeding and extensive vascular repair, capped inflated cylinders or malleable cylinders may be left in situ. If the bleeding is controlled and the patient stabilised, proceeding with the inflatable implant with the reservoir in an alternative position is a viable option.

## 5 CONCLUSION

Reservoir‐related injuries during IPP implantation are uncommon but potentially serious. Safe reservoir placement relies on anticipation, preparation, early recognition and decisive intraoperative management. For the practising andrologist, the central principle is that prevention is preferable to salvage.

In practical terms, this means the following:

1. Prevention. In men with hostile pelvic anatomy, including prior pelvic surgery, radiotherapy or mesh hernia repair, select HSM, extraperitoneal, or other ectopic reservoir sites as the primary strategy to avoid complications altogether.
2. Preparation. Identify high‐risk patients preoperatively, ensure bladder decompression, position the patient appropriately and confirm the availability of additional expertise and equipment, including general or vascular surgical support, when required.
3. Perception. Maintain a high index of suspicion for injury when there is loss of resistance, clear fluid in the operative field, sudden bleeding or entry into the peritoneum. Use intraoperative cystoscopy liberally when bladder injury is suspected.
4. Proaction. Manage bladder injuries with prompt confirmation, secure closure and reservoir relocation or staged implantation. Control vascular injuries with immediate haemostasis, early vascular input and a low threshold to abandon implantation if bleeding cannot be rapidly controlled. Treat bowel injuries by immediately halting implantation and involving general or colorectal surgery.

This guide provides a practical framework for managing reservoir‐related complications during penile prosthesis surgery. By prioritising prevention over salvage, anticipating risk, preparing adequately and adapting to intraoperative findings, surgeons can minimise morbidity, manage complications effectively and preserve the high satisfaction rates that underpin modern penile prosthesis implantation.

## AUTHOR CONTRIBUTIONS

All authors contributed to material preparation and write up of the paper. All authors reviewed and edited the final manuscript.

## CONFLICT OF INTEREST STATEMENT

The authors declare that they have no competing financial interests, including grants, royalties, licences, consulting fees, payments or honoraria (including for attending meetings), stock ownership or patents.
